# Supplementary material for: Aurivillius Oxides Nanosheets-Based Photocatalysts for Efficient Oxidation of Malachite Green Dye
Source: Int J Mol Sci. 2022 May 12;23(10):5422. doi: 10.3390/ijms23105422 (PMC9140923; doi:10.3390/ijms23105422)
Supplement: Supplementary file 1 [file ijms-23-05422-s001.zip › ijms-1682923-supplementary.pdf]

Supporting information

Aurivillius Oxides Nanosheets based photocatalysts for efficient oxidation of malachite green dye

*David A. Collu,[1]§ Cristina Carucci,[1,2]§ Marco Piludu,[2,3] Drew F. Parsons,[1,2] and Andrea Salis[1,2]**

[1] *Department of Chemical and Geological Sciences, University of Cagliari, Cittadella Universitaria,*
S.P. 8 km 0.700, 09042 Monserrato (CA), Italy

[2] *Consorzio Interuniversitario per lo Sviluppo dei Sistemi a Grande Interfase (CSGI), via della Lastruccia 3, 50019, Sesto Fiorentino (FI), Italy*

[3] *Department of Biomedical Sciences, University of Cagliari, S.P. 8 km 0.700, 09042 Monserrato (CA), Italy*

* *Correspondence: asalis@unica.it*

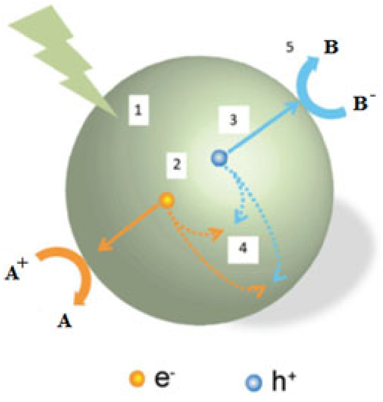

Scheme S1. Schematic diagram of the generic process of photocatalysis, with an oxidant **A** and a reductant **B**. Photon absorption (1) creates two opposed charge carriers: electrons and vacancies. These must separate (2), migrate (3) and reach the surface reaction sites, where the redox processes take place (5). Photogenerated carrier recombination (4) is the main cause of reduced efficiency of the whole process. Longer-wavelength electromagnetic radiation can be emitted in a radiative decay. Alternatively, energy is wasted as thermal agitation (nonradiative decay). Adapted from Ref. [1]

XRD measurements (Fig. S1A) of BMO synthesis highlights the increase in plot resolution which is obtained by longer hydrothermal steps in the synthesis process as reported in outline of synthetic methods and characterizations. This could be due to an increase in crystallite mean size and/or increase in BMO nanosheet dimensions. Overall, XRD shows that BMO synthesis is sensitive to the duration of heating, whereas BWO showed no increase, or a slight decrease, in NS size after 3 hours in the oven at 140 °C (Fig. 1B).

TGA analysis of BMO samples was carried out (Fig. S1B). TGA exhibit some similar features through all the materials. It is possible to notice that the longer duration of the hydrothermal step reduces contamination through the BMO series. BWO and BMO-24 residual PVP content was very small, around 3 %. The contamination percentages for all BMO materials follow the order BMO-0 (8 %) > BMO-3 (3 %) > BMO-24 (7 %) confirming BMO-24 as the catalyst with the lowest contamination.

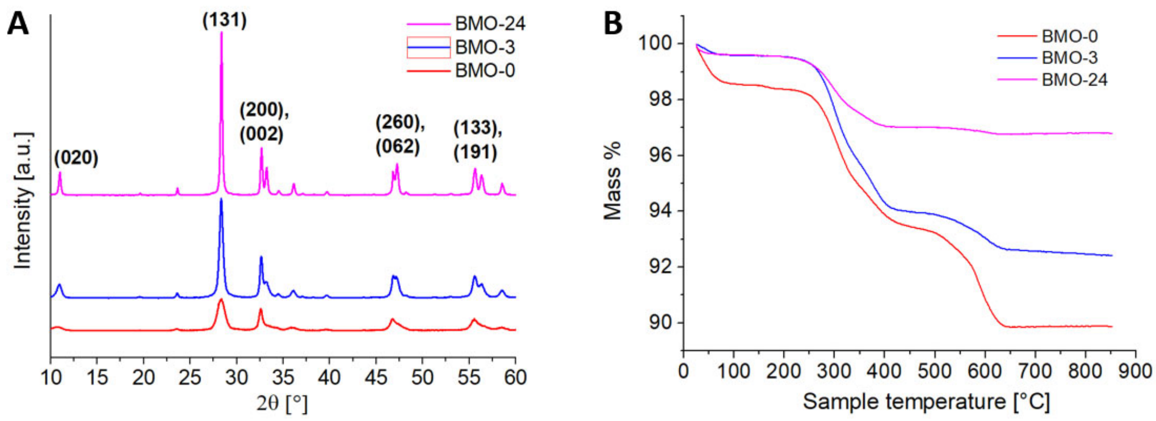

Figure S1. XRD **(A)** and TGA **(B)** of BMO series of photocatalysts

DLS (Fig. S3B) also shows that BMO NSs size clearly become larger after time in autoclave going from 100 ± 7 nm of BMO-0 to 160 ± 9 nm according with XRD data. After the first three hours in the autoclave, however, the average size of NSs does not change significantly (160 nm in BMO-3 and 145 nm in BMO-4 are comparable), but the polydispersity index (PdI) increases. According to these observations, the PdI never exceeded 0.3 for all materials, except for BMO-24, where it reached 0.5. This value gives a rough indication of the quality of the monodispersion (Table S1).

Table S1. Preparation methods used for BMO.

Sample	Synthesis method	Refs
$BMO-0$	$Na_2MoO_4 \cdot 2H_2O$ added at the beginning of every step. 50 °C under stirring.	2
$BMO-3$	Reagents added at the beginning. Hydrothermal reaction, 140 °C for 3 hours.	This work
$BMO-24$	. Reagents added at the beginning. Hydrothermal reaction, 140 °C for 24 hours	This work

BMO showed a substantial improvement with the modifications applied in its preparation. The second synthetic method for BMO, which was developed in this work, involved a hydrothermal step, with the aim of obtaining a purer and more functional photocatalyst in a shorter time. BMO-24 as synthesized in the present work showed fewer organic impurities (seen with TGA in Fig 2B). Because

of that it was used for all further characterizations and catalytic tests and from now on will be referred in the text simply as BMO.

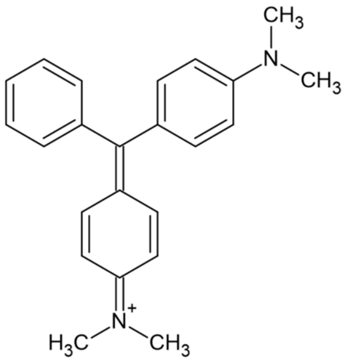

Figure S2. Chemical structure of malachite green in its cationic form (green as solid crystal, cyan in aqueous solution).

Table S2. Peaks position used for crystallite size calculation through Scherrer theory.

**Peak positions (2θ; °)**
10.03, 39.51
28.36; 58.60
10.70; 28.32
10.92; 28.34
10.98; 28.37

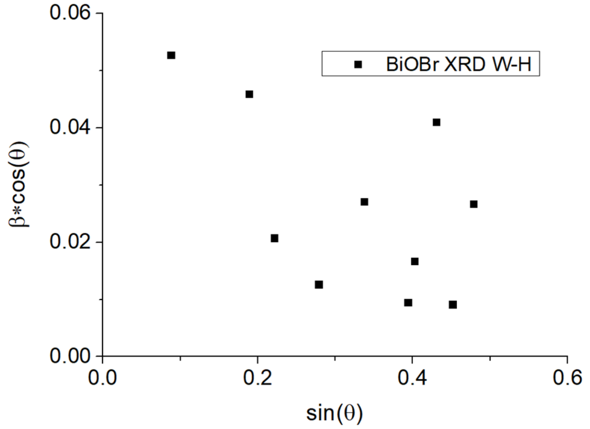

Figure S3. Crystallites size analysis of BiOBr.

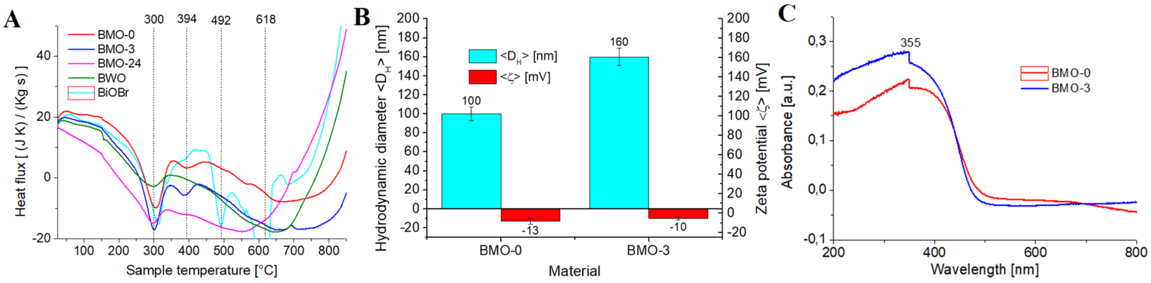

Figure S4. A) DSC of BiOBr, BWO, BMO-0, BMO-3 and BMO-24 DSC **B)** DLS and ELS of BMO-0 and BMO-3; **C)** DRS of BMO-0 and BMO-3.

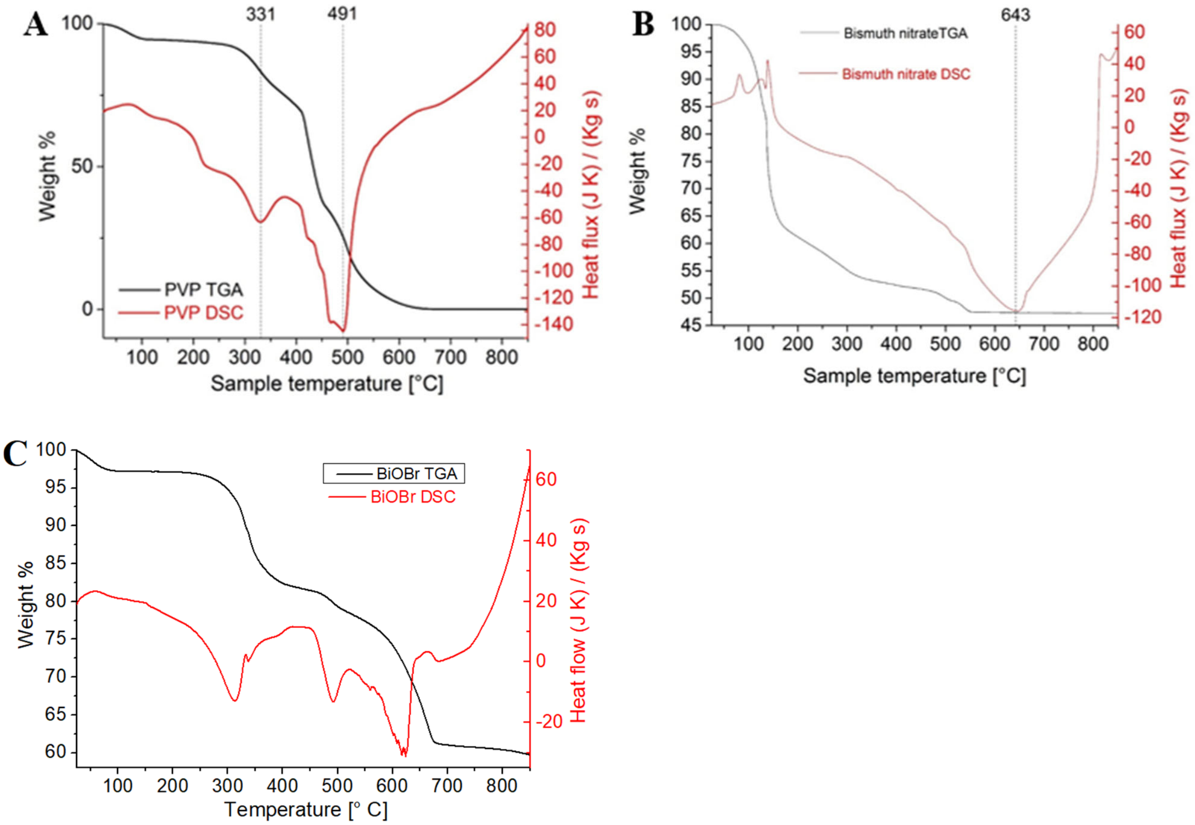

Figure S5. A) TGA and DSC of pure PVP. **B)** TGA and DSC of pure $Bi(NO_3)_3{\cdot}5H_2O$. **C)** TGA and DSC of BiOBr.

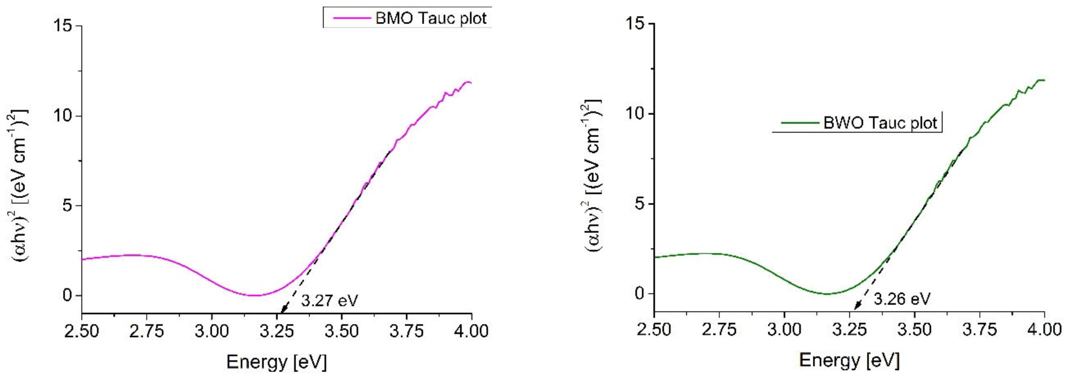

Figure S6. Tauc plot for **A)** BMO and **B)** BWO materials. Arrows represent the band gap energy potential E_g.

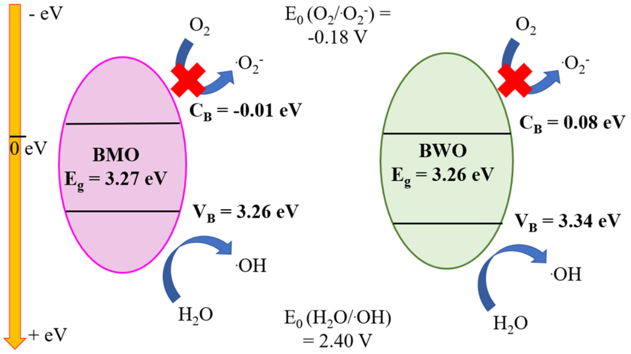

Figure S7. Hypothetical mechanism of production of hydroxyl radical by BMO and BWO photo-catalysts. Potentials of relevant redox couples are indicated. Scheme adapted by Ref . [3]

$$MG(\%) = (\frac{A_0 - A}{A_0} \times 100)$$

Equation. S1 Percentage of MG removal (%) calculated as percentage of absorbance decrease. A_0: Absorbance at 617 nm of MG starting solution, A: Absorbance at 617 nm of MG solution after BWO/BMO degradation.

Photocatalytic experiments were implemented to quantify the amount of dye degraded by BWO and BMOs. Four glass vessels were used, labeled **1**, **2**, **3**, **4** respectively. Their content and treatment in terms of light irradiation is summarized in Table S3.

Table S3: Photocatalytic tests design.

Sample	*Components*	*Light irradiation*
1	*MG/BWO*	*Yes*
2	*MG/BWO*	*No*
3	*MG*	*Yes*
4	*MG*	*No*

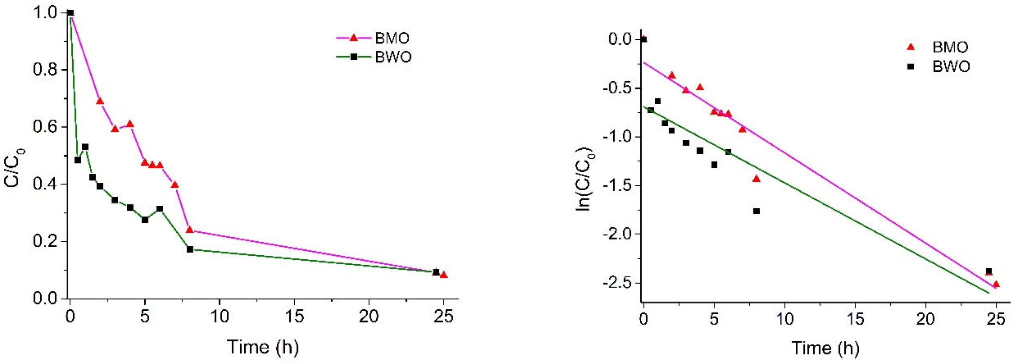

Figure S8. A) C/C0 and **B)** ln(C/C0) MG kinetic values vs irradiation time fitted with Langmuir-Hinshelwood model.

Table S4. Kinetic parameters from the Langmuir- Hinshelwood model.

Photocatalyst	k x10^{-2} [min^{-1}]	Equation	R^2
**BWO**	7.81	y = - 0.07812x - 0.6906	0.75
**BMO**	9.27	y = - 0.09269x - 0.23672	0.95

References

(1) Zhu, S.; Wang, D. Photocatalysis: Basic Principles, Diverse Forms of Implementations and Emerging Scientific Opportunities. *Adv. Energy Mater.* **2017**, *7* (23), 1–24. https://doi.org/10.1002/aenm.201700841.

(2) Di, J.; Zhao, X.; Lian, C.; Ji, M.; Xia, J.; Xiong, J.; Zhou, W.; Cao, X.; She, Y.; Liu, H.; Ping, K.; Pennycook, S. J.; Li, H.; Liu, Z. Atomically-Thin Bi 2 MoO 6 Nanosheets with Vacancy Pairs for Improved Photocatalytic CO 2 Reduction. **2019**, *61* (March), 54–59. https://doi.org/10.1016/j.nanoen.2019.04.029.

(3) Omrani, N.; Nezamzadeh-Ejhieh, A.; Alizadeh, M. Brief Study on the Kinetic Aspect of Photodegradation of Sulfasalazine Aqueous Solution by Cuprous Oxide/Cadmium Sulfide Nanoparticles. *Desalin. Water Treat.* **2019**, *162*, 290–302. https://doi.org/10.5004/dwt.2019.24352.
